# Exploring the multifaceted functions of APPL in metabolism and memory using *Drosophila melanogaster*

**DOI:** 10.1016/j.mocell.2024.100163

**Published:** 2024-11-26

**Authors:** Dharmendra Kumar Nath, Youngseok Lee

**Affiliations:** Department of Bio and Fermentation Convergence Technology, Kookmin University, Seoul 02707, Republic of Korea

**Keywords:** Alzheimer’ disease, Ascorbic acid, Cognition, Curcumin, Metformin

## Abstract

Amyloid precursor protein (APP) is a single-pass transmembrane protein abundantly expressed in the central nervous system and implicated in familial Alzheimer’s disease, a progressive neurodegenerative disorder that impairs memory. Here, we investigated the role of amyloid precursor protein-like (APPL) using the model organism *Drosophila melanogaster*. In this study, *Appl* null mutants exhibited a reduced lifespan under normal conditions and increased triglyceride levels, which were mitigated by metformin treatment. Additionally, taste-associative memory impairment in *Appl*^*d*^ mutants suggested APPL’s role in memory formation, which was restored by curcumin supplementation. The *Appl*^*d*^ mutants also displayed reduced climbing ability, which was improved by supplementation with vitamins C (ascorbic acid) and B_2_ (riboflavin). These findings suggest that APPL is involved in metabolic regulation, cognition, climbing activity, and aging in *Drosophila melanogaster*.

## INTRODUCTION

Alzheimer’s disease (AD) is an irreversible, progressive, and degenerative brain disorder that has recently emerged as the most prevalent type of dementia (Auld et al., 2002). AD is characterized by the accumulation of amyloid plaques and neurofibrillary tangles in brain tissues (Shipton et al., 2011). The proteolytic processing of the integral membrane protein amyloid precursor protein (APP) produces amyloid-beta peptides, which aggregate into amyloid plaques in the brain (Luo et al., 1992, Wilquet and De Strooper, 2004). The excessive accumulation of amyloid plaques impedes neuronal communication and elicits inflammatory reactions, consequently leading to neuronal damage and cell death (Papuć and Rejdak, 2020, Yu et al., 2018). Additionally, neurofibrillary tangles alter intracellular transport and structural integrity, resulting in synaptic dysfunction and neuronal cell death. In addition to its link to the onset and progression of AD, APP is known to be involved in numerous physiological processes, including G-protein-coupled receptor signaling, cell adhesion, neurite outgrowth, and synaptogenesis (Darbandi et al., 2020, Zheng and Koo, 2006).

In mammals, 2 paralogs of APP, APP-like protein-1 and -2 (APLP1 and APLP2), are highly conserved (Jacobsen and Iverfeldt, 2009). Additionally, 2 invertebrate model systems have been found to harbor APP homologs: the amyloid precursor protein-like (APPL) in *Drosophila melanogaster* and the amyloid protein–like protein 1 (APL-1) in *Caenorhabditis elegans* (Daigle and Li, 1993). APPL is expressed exclusively in the nervous system, including most developing and mature neurons, and is involved in axonal transport, neuronal development, formation of synaptic boutons, and the neuronal response to brain injury (Ashley et al., 2005, Goguel et al., 2011, Gunawardena and Goldstein, 2001, Li et al., 2004). APP participates in vital biological functions related to morphogenesis and tissue remodeling, acting both as a contact receptor and a diffusible factor (Gralle and Ferreira, 2007). Mice lacking APP and its homolog APP-like protein 2 exhibit abnormal apposition of presynaptic marker proteins with postsynaptic cholinergic receptors and increased nerve terminal sprouting (Wang et al., 2005). APPL overexpression in *Drosophila* markedly increases the number of synaptic boutons and structural modifications in synapses, while a reduction in the number of boutons is observed in an *Appl* null mutant (Torroja et al., 1999). APPL has been shown to be essential for long-term memory and is highly expressed in mushroom bodies (MBs), a specific brain region involved in olfactory memory (Goguel et al., 2011). In vivo models of progressive degeneration indicate that APP/APPL plays a neuroprotective role, with the absence of APPL leading to neurodegeneration in flies. In *C*. *elegans*, loss of APL-1 affects various developmental processes such as morphogenesis, molting, and larval death, with neuronal expression of the extracellular domain of APL-1 being able to recapitulate larval lethality (Hornsten et al., 2007).

This study sought to explore additional functions of APPL. Our findings revealed that APPL is involved in lipid metabolism, memory formation, climbing ability, and the aging process. Furthermore, we found that metformin can reduce excessive triglyceride (TAG) levels, curcumin can improve taste-associative memory impairment, and dietary supplementation with ascorbic acid and riboflavin can enhance climbing ability in APPL mutants.

## MATERIALS AND METHODS

### *Drosophila* Strains

All flies were maintained at 25°C under a 12-hour light/12-hour dark photoperiod. Wild-type (*w*^*1118*^) flies were used as a control strain. *Appl*^*d*^ (BDSC 43632), *Appl*-*GAL4* (BDSC 32040), *UAS*-*Appl* (BDSC 29862), *UAS-Kir2.1* (BDSC 6596), *MB*-*GAL4* (BDSC 204), and *Akh*-*GAL4* (BDSC 25684) were obtained from the Bloomington *Drosophila* stock center. *Dh44*-*GAL4* (BDSC 1453) was provided by Y. Kim (GIST). *dilp2*-*GAL4* (KDRC 200), *UAS*-*AMPK*^*R*^ (KDRC 10098), and *UAS*-*AMPK*^*TD*^ (KDRC 10099) were acquired from the Korea *Drosophila* Research Center. *cg-GAL4* and *Dmef-GAL4* were kindly provided by S. Hyun (Chung-Ang University).

### Chemical Sources

Trehalase from porcine kidney (cat. # 9025-52-9), amyloglucosidase (cat. # 9032-08-0), the glucose (HK) assay kit (cat. # GAHK-20-1KT), curcumin (cat. # 1386-10G), ascorbic acid (cat. # A7506-100G), riboflavin (cat. # R9504-25MG), metformin (cat. # D150959), and Nile red (cat. # N-3013) were purchased from Sigma-Aldrich Co. The Pierce BCA protein assay kit (cat. # 23225) and the LiquiColor TAG test kit (cat. # 2100-225) were purchased from Thermo Fisher Scientific and Stanbio Laboratory, respectively.

### Hemolymph Glucose Measurement

Hemolymph glucose was measured as described previously (Dhakal et al., 2022, Dus et al., 2011). Hemolymph samples were collected from 6-day-old flies. The flies were punctured in the thorax using a fine injection needle and transferred to 0.5-ml tubes with a punctured base. The flies were placed on their sides to prevent leakage from the genital tract. Afterward, the tubes were placed inside 1.5-ml microfuge tubes and centrifuged at 4°C for 5 minutes at 2,800 *g*. Next, 0.5 µl of hemolymph was added to 14.5 µl of PBS and maintained at 70°C for 5 minutes. Then, 100 µl of glucose reagent (Sigma: 3293 VER) was added and incubated at 37°C for 12 hours. The assays were conducted using a commercial glucose (Hexokinase: HK) assay kit and total glucose was measured at 340 nm. Glucose levels were quantified by comparing the values with a glucose standard curve.

### Trehalose and Glucose Measurements in Whole Adult Flies

Whole-body glucose and trehalose levels were measured in adult flies as described previously (Dhakal et al., 2022, Meunier et al., 2007). Briefly, ten 6- to 10-day-old and 30-day-old males were collected, weighed, and crushed in 250 µl of 0.25 M Na_2_CO_3_ buffer. The homogenates were then incubated in a water bath (95°C) for 5 minutes to inactivate all enzymes. Next, 600 µl of 0.25 M sodium acetate and 150 µl of 1 M acetic acid (pH 5.2) were added to the samples, after which the mixtures were centrifuged at 12,500 *g* for 10 minutes at 24°C. Afterward, 200 µl of supernatant was transferred to a new microfuge tube, followed by 2 µl porcine kidney trehalase (Sigma: T8778 UN). The mixture was then incubated overnight at 37°C to convert trehalose into glucose. Next, 1 ml of glucose hexokinase solution (Sigma: GAHK-20) was added to 100 µl of the sample and incubated for 20 minutes at 37°C. Optical density values were measured at 340 nm. Finally, total glucose and trehalose levels were calculated using a standard glucose curve generated through similar reactions with standard trehalose and glucose.

### Glycogen Measurements

Tissue glycogen levels from whole-body extracts of adult flies were quantified as previously described (Dhakal et al., 2022, Dus et al., 2011). Briefly, five 6- to 10-day-old and 30-day-old males were weighed and homogenized in 100 µl of ice-cold 1× PBS. The homogenates were kept at 70°C for 5 minutes to inactivate all metabolic enzymes. The samples were then centrifuged at 12,500 *g* for 3 minutes at 4°C, after which 20 µl of the supernatant was transferred to 1.5-ml microfuge tubes and diluted with 1× PBS to a 1:3 ratio. An amyloglucosidase dilution was prepared by mixing 1.5 µl of amyloglucosidase (Sigma A1602) suspension in 998.5 µl 1× PBS. Finally, a 20-µl aliquot of the diluted sample was added to 20 µl of the diluted amyloglucosidase solution, as well as to the glycogen standard. Both the glycogen standard and test samples were then incubated at 37°C for 1 hour. Total glucose was measured at 340 nm using a commercial glucose (HK) assay reagent (Sigma, G3293 VER). The glycogen level was quantified by comparing it with a standard curve plotted from standard glycogen samples.

### TAG Level Measurement

TAG levels were quantified as described previously, with some modifications, using a LiquiColor TAG Test kit (cat. # 2100-225, Stanbio Laboratory) (Sang et al., 2021). Ten 6- to 10-day-old male flies were weighed and crushed in 1 ml of PBST (1× PBS and 0.2% Triton X-100). The homogenate was incubated at 70°C for 5 minutes and centrifuged for 3 minutes at 9,500 *g*. Next, 100 µl of the supernatant was transferred to a 1.5-ml Eppendorf tube and mixed with 1 ml of Stanbio LiquiColor TAG Test kit reagent or 1 ml of deionized water as a control. The reaction mixture was kept at 37°C for 15 minutes. Finally, the absorbance of the sample solution was measured at 500 nm using a spectrophotometer, and the TAG level was calculated based on a standard calibration curve.

### Protein Level Measurement

Protein assays were performed as previously described (Nath et al., 2024) using the Pierce BCA Protein Assay Kit, with some modifications. Briefly, ten 6- to 10-day-old and 30-day-old male flies were weighed and crushed in 1 ml of PBST (1× PBS and 0.2% Triton X-10) and incubated at 70°C for 5 minutes. The homogenate mixture was then centrifuged for 3 minutes at 9,500 g, after which 300 µl of supernatant was mixed with 600 µl of Pierce BCA Protein Assay Kit reagent (UF289330). After a 30-minute incubation period at 37°C, the absorbance of the samples was measured at 530 nm using a spectrophotometer and compared with a standard calibration curve for quantification.

### Survival Assay

Survival experiments were conducted as previously described (Lee et al., 2018b). Twenty male and female flies were evaluated separately to measure normal survival time on standard cornmeal food. The flies were monitored and counted every 24 hours, after which the live flies were transferred to vials containing new food. To measure survival under starvation conditions, twenty 3- to 4-day-old male and female flies were fed with 1% agar food. The flies were monitored and counted every 12 hours, after which they were transferred to fresh vials with the same food supply. The experiments continued until all the flies had died.

### Immunohistochemistry

Immunohistochemistry analyses were conducted as previously described (Dhakal et al., 2022). Freshly dissected tissues (brain and intestine) were first transferred to 24-well tissue culture plates (Costar Corp.) on ice containing 940 µl of fixing buffer (1 mM EGTA, 0.1 M Pipes pH 6.9, 2 mM MgSO_4_, 1% Triton X-100, and 150 mM NaCl). Then, 60 µl of formaldehyde (37%) was added to the wells and mixed immediately before adding the tissue. The required number of tissue samples, dissected within 15 minutes, were fixed and incubated for 30 minutes. The samples were then washed with wash buffer (1× PBS, 0.1% saponin) 3 times (15 minutes each) and blocked with 1 ml blocking buffer (1× PBS, 0.1% saponin, and 5 mg/ml BSA) at 4°C for 4 to 8 hours.

For immunostaining, primary antibodies {mouse anti-GFP (1:1,000, Molecular Probes, cat. # A11120)} were added to the samples at 4°C for 18 hours. The samples were then washed with wash buffer 3 times (15 minutes each) and then incubated with secondary antibody {(1:200) goat anti mouse Alexa Fluor 488 (cat. # A11029)} at 4°C for 4 hours. Finally, the samples were washed 3 times, stored in 1.25× PDA (187.5 mM NaCl, 37.5% glycerol, and 62.5 mM Tris pH 8.8), and kept at 4°C for more than 1 hour. The samples were then mounted and examined using a Leica Stellaris 5 Confocal Microscope.

### Nile Red Staining

A stock solution of Nile red (Sigma N-3013), a dark purplish-red powder, was prepared in acetone (1,000 µg/ml) and kept in a tightly sealed, lightproof container at 4°C. Briefly, 6- to 10-day-old male flies were fixed in a sagittal position on a glass slide and submerged in a 1× PBS solution. Fat bodies (FBs) were gently dissected from the dorsal abdominal region along with the thorax or the gut under a stereomicroscope. The dissected tissues were then fixed with 4% formaldehyde solution for 15 minutes at room temperature. The fixed tissues were gently washed 3 times with 1× PBS (5 minutes per wash). Nile red (1:1,000 dilution) was then added to the tissue samples for 5 minutes. Finally, the stained tissues were washed with 1 ml 1× PBS and mounted in 50% glycerol on a glass slide. Comparisons were made between segments 2 and 3 of the abdomen across all samples to ensure consistency and accuracy.

### Climbing Assay

Climbing assays were conducted as described previously (Madabattula et al., 2015), with some modifications. Twenty male flies were collected, anesthetized with CO_2_, and kept in a food vial for 24 hours before the experiment. A cylindrical glass vial was marked 17.5 cm from the base. The collected flies were then transferred to the cylindrical vial, which was then plugged with a cotton plug to prevent any flies from escaping. A camera was set up to record the experiment. The vial was tapped lightly 3 to 4 times against a pad to encourage the flies to move to the bottom of the vial. One-minute video clips were then recorded, and the number of flies crossing the 17.5-cm mark was quantified every 10 seconds.

### Learning and Memory Assay

Learning and memory assays were performed as previously described (Poudel and Lee, 2018, Sang et al., 2024). Briefly, 6- to 10-day-old flies were collected and starved for 18 to 20 hours before the experiment. The flies were kept in an empty glass vial and anesthetized on ice. Fifteen to 20 flies were fixed on a glass slide using nail polish. The flies were then kept in an incubator at 25°C for 30 minutes to recover. In the pretest phase, water stimuli were applied to the labellum of each fly. A 500 mM sucrose stimulus was applied to the leg of each labellum. Only flies that displayed a proboscis extension response were selected for the training phase. During the training phase, a 500 mM sucrose stimulus was applied to the leg, while a 50 mM caffeine stimulus was simultaneously applied to the labellum. The training was repeated 15 times, and the number of flies exhibiting a positive proboscis extension response was recorded. In the test phase, a 500 mM sucrose stimulus was applied to the labellum at 0, 5, 15, 30, 45, and 60 minutes, and the number of flies that displayed a proboscis extension response was recorded.

### Reverse Transcription Polymerase Chain Reaction and Quantitative Reverse Transcription Polymerase Chain Reaction

Briefly, ten 6- to 10-day-old male flies were selected for whole-body analysis. Total RNA was extracted using the TRIzol reagent (cat. # 1556018) followed by DNase treatment (cat. # M610A). cDNA was synthesized using the AMV reverse transcriptase system (cat. # M510A). To perform reverse transcription polymerase chain reaction, we used the *Appl* primers: 5′-ATC CAC AAC GCC TCC CGA TG-3′ (forward) and 5′-CTA GGC GCA TGT CTG GTA-3′ (reverse). Quantitative reverse transcription polymerase chain reaction (qRT-PCR) assays were performed as previously described (Lee et al., 2009, Nath et al., 2024, Yoshinari et al., 2021). The experiments were conducted under sated conditions. qRT-PCR experiments were carried out using a Bio-Rad CFX system. The mRNA expression levels of each gene were assessed using Takara TB Green Premix according to the manufacturer’s instructions. Relative gene expression was calculated using the 2^–∆∆Ct^ method. Three biological samples were used, and transcript levels were normalized to the *D. melanogaster* housekeeping gene *tubulin*. The following primer pairs were used for the experiments: *Appl*, 5′-ATT CCA ATC TGA CGC CCT GC-3′ (forward) and 5′-ATC GGT TTT GAA GTG CTT GG-3′ (reverse); *acc*, 5′-ACG AGG GCG AGC AGC GTT AC-3′ (forward) and 5′-TAG GGC GAC TTG GTG GGC AT-3′ (reverse); *bmm*, 5′-ATG ACT TCG GAC TTC TTC AGG G-3′ (forward) and 5′-CCA ATT CAG ATG GAA GAG CTG-3′ (reverse); *desat1*, 5’-AAG CCG GTG CCC AGT CCA TC-3’ (forward) and 5’-ATG GTC GCG AGC CCA ATG GT-3′ (reverse); and *tubulin*, 5′-TCC TTG TCG CGT GTG AAA CA-3′ (forward) and 5′-CCG AAC GAG TGG AAG ATG AG-3' (reverse).

### Statistics and Reproducibility

*D. melanogaster* was used as the model organism in this study. Male flies were predominantly used for the experiments unless otherwise specified. All experiments were conducted under controlled laboratory conditions. The number of replicates for each experiment was determined based on prior research to ensure sufficient sample size for reliable and repeatable results. No data points were excluded from the analysis. The data points for each genotype represent values from individual replicates. The error bars in the figures denote the standard error of the mean (SEM). qRT-PCR data were analyzed using C_T_ values. Comparisons between multiple experimental groups were made using single-factor ANOVA followed by Scheffe’s post hoc test. Pairwise comparisons were performed using Student’s *t*-test. Survival curves for each group were analyzed using the Kaplan-Meier method and compared statistically using log-rank tests. Statistical significance is indicated by asterisks in the figures (*P < .05, **P < .01). All statistical analyses were conducted using the Origin Pro 8 software for Windows (ver. 8.0932; Origin Lab Corporation).

## RESULTS

### *Appl* Mutation Results in Shorter Lifespan, Increased Lipid Deposition, and Enhanced Starvation Resistance

**Fig. 1:**
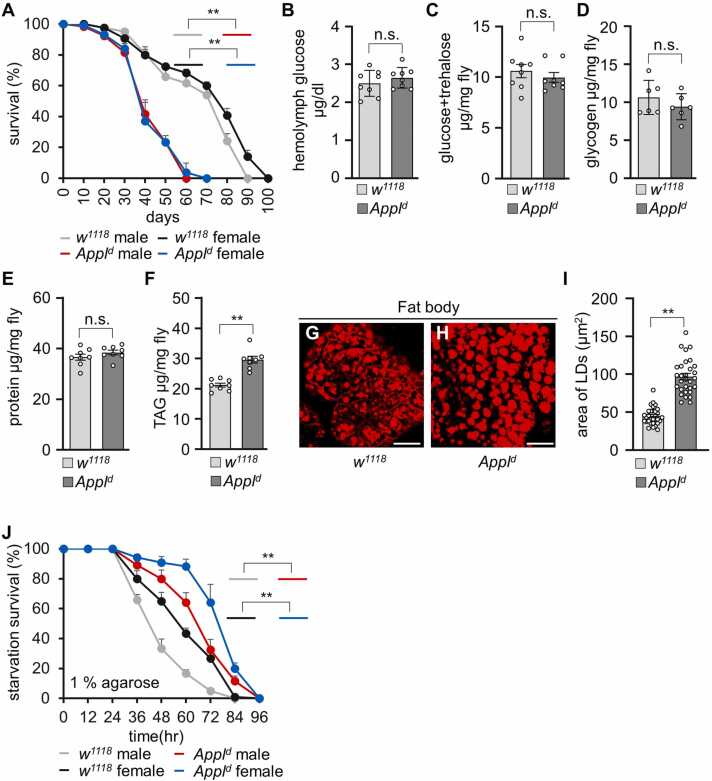
*Appl*^*d*^ mutants exhibit reduced lifespan, increased lipid level, and starvation resistance. (A) Survival time under standard cornmeal food (*n* = 6). (B) Hemolymph glucose levels (*n* = 8). (C) Glucose and trehalose levels (*n* = 8). (D) Glycogen levels (*n* = 6). (E) Protein levels (*n* = 8). (F) TAG levels (*n* = 8). (G, H) Nile red staining of LDs in the fat body of adult *w*^*1118*^ and *Appl*^*d*^ flies. Scale bar = 50 µm. (I) Measurement of LD areas in (G, H) (*n* = 3). (J) Measurement of starvation resistance in 1% agarose (*n* = 6). All values are presented as means ± SEM. Comparisons between multiple experimental groups were performed using single-factor ANOVA followed by Scheffe’s post hoc test. The survival curves in A were analyzed for each group using the Kaplan-Meier method and compared statistically using log-rank tests. The asterisks indicate significant differences from the controls (**P < .01). Each dot indicates the distribution of individual sample values.

We first verified the *Appl*^*d*^ is a null allele using reverse transcriptase polymerase chain reaction analysis (Fig. S1A). The expected band was observed only in wild-type cDNA and not in *Appl*^*d*^ cDNA. To investigate the impact of APPL on lifespan, we compared the survival rates of both male and female *Appl*^*d*^ and control (*w*^*1118*^) flies on standard cornmeal food. *Appl*^*d*^ males and females exhibited significantly shorter lifespans compared with control flies (Fig. 1A). Specifically, the LT_50_ for male control and *Appl*^*d*^ flies was 69.27 ± 3.09 days and 40.14 ± 2.45 days, respectively. For female control and *Appl*^*d*^ flies, the LT_50_ was 75.19 ± 1.80 days and 40.93 ± 2.15 days, respectively. Energy homeostasis is known to be directly linked to lifespan. Therefore, carbohydrate, lipid, and protein levels were assessed to understand the role of APPL in energy balance. We used 6- to 10-day-old male flies to compare nutrient levels. The carbohydrate levels in *Appl*^*d*^ flies were similar to those of the controls. Circulatory glucose in the hemolymph of control and *Appl*^*d*^ flies was 2.50 ± 0.12 µg/dl and 2.65 ± 0.09 µg/dl, respectively (Fig. 1B). Whole-body trehalose and glucose levels were 10.78 ± 0.65 µg/mg fly and 10.15 ± 0.48 µg/mg fly for control and *Appl*^*d*^ flies, respectively (Fig. 1C). In animals, carbohydrates are stored as glycogen. The whole-body glycogen levels in control and *Appl*^*d*^ were 10.81 ± 0.92 and 9.59 ± 0.71 µg/mg fly, respectively (Fig. 1D). Whole-body protein levels were not significantly different from control (Fig. 1E). However, lipid levels, stored as TAGs in the FB, were significantly higher in *Appl*^*d*^ (33.68 ± 0.25 µg/mg fly) compared with the controls (27.02 ± 0.62 µg/mg fly) (Fig. 1F). We also examined 30-day-old flies to verify carbohydrate and protein levels and found no difference in carbohydrate and protein levels in aged flies (Fig. S1B-D). Next, we dissected the FB from the abdomen and stained it with Nile red to quantify lipid droplet (LD) areas (Fig. 1G and H). LDs in *Appl*^*d*^ were much larger than in control flies (Fig. 1I). Under starvation or nutrient-deprived conditions, stored lipids are used as an energy source. Our findings revealed that *Appl*^*d*^ flies with higher TAG levels survived longer under starvation compared with the control flies (Fig. 1J). These findings suggest that APPL is crucial for lipid metabolism regulation, which in turn impacts lifespan.

### *Appl*^*d*^ Defects in Lipid Deposition Can Be Rescued by the Wild-type Appl and Activated Form of AMP-activated Protein Kinase

**Fig. 2:**
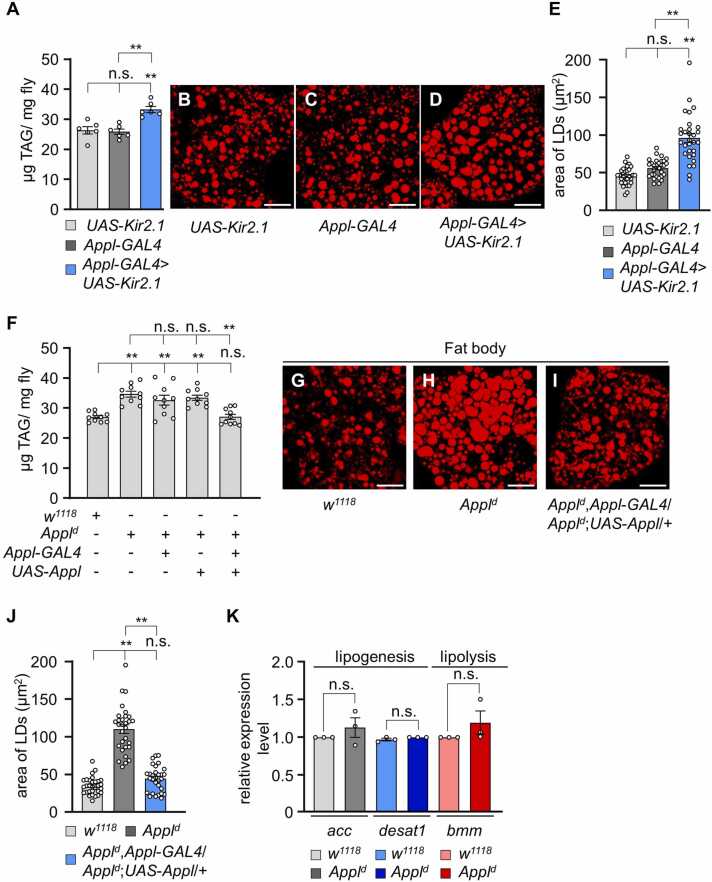
APPL regulates lipid metabolism. (A) Measurement of TAG level in *UAS*-*Kir2.1*, *App*l-*GAL4*, and *Appl*-*GAL4*/+;*UAS*-*Kir2*.*1*/*+* (*n* = 6). (B-D) Nile red staining of lipid droplets in the fat body of *UAS*-*Kir2*.*1*, *Appl*-*GAL4*, and *Appl*-*GAL4*/+;*UAS*-*Kir2*.*1*/+. Scale bars = 50 µm. (E) Measurement of LD area in the fat body of *UAS*-*Kir2*.*1*, *Appl*-*GAL4*, and *Appl*-*GAL4*/+;*UAS*-*Kir2*.*1*/*+* (*n* = 3). (F) Measurement of TAG level in *w*^*1118*^, *Appl*^*d*^, *Appl*^*d*^,*Appl*-*GAL4*, *Appl*^*d*^;*UAS*-*Appl*, and *Appl*^*d*^,*Appl*-*GAL4*/*Appl*^*d*^;*UAS*-*Appl*/+ (*n* = 10). (G-I) Nile red staining of LDs in the fat body of *w*^*1118*^, *Appl*^*d*^, and *Appl*^*d*^,*Appl*-*GAL4*/*Appl*^*d*^;*UAS*-*Appl*/+. Scale bars = 50 µm. (J) Measurement of LD area in the fat body of *w*^*1118*^, *Appl*^*d*^, and *Appl*-*GAL4*/*Appl*^*d*^;*UAS*-*Appl*/+ (*n* = 3). (K) Quantification of the expression level of lipogenic (*acc* and *desat1*) and lipolytic (*bmm*) genes under sated conditions (*n* = 3). All values are reported as means ± SEM. Comparisons between multiple experimental groups were conducted via single-factor ANOVA with Scheffe’s post hoc test. The asterisks indicate significant differences from the controls or the indicated genotypes (**P < .01). Each dot represents the distribution of individual sample values. (+) and (−) indicate the presence or absence of the indicated transgenes, respectively.

To further investigate the regulatory role of APPL in lipid metabolism, we inactivated *Appl*-*GAL4* by expressing the inwardly rectifying potassium channel *UAS*-*Kir2.1* (Hodge, 2009). This inactivation led to an increase in whole-body TAG levels from 26.13 ± 1.27 µg/mg to 33.63 ± 14 µg/mg (Fig. 2A). Similarly, we also stained the lipids in the FB with Nile red and measured the area of LDs (Fig. 2B-D). The LDs in *Appl*^*d*^ flies were significantly larger than those in the controls (Fig. 2E), indicating that *Appl* activity is crucial for maintaining lipid homeostasis.

**Fig. 3:**
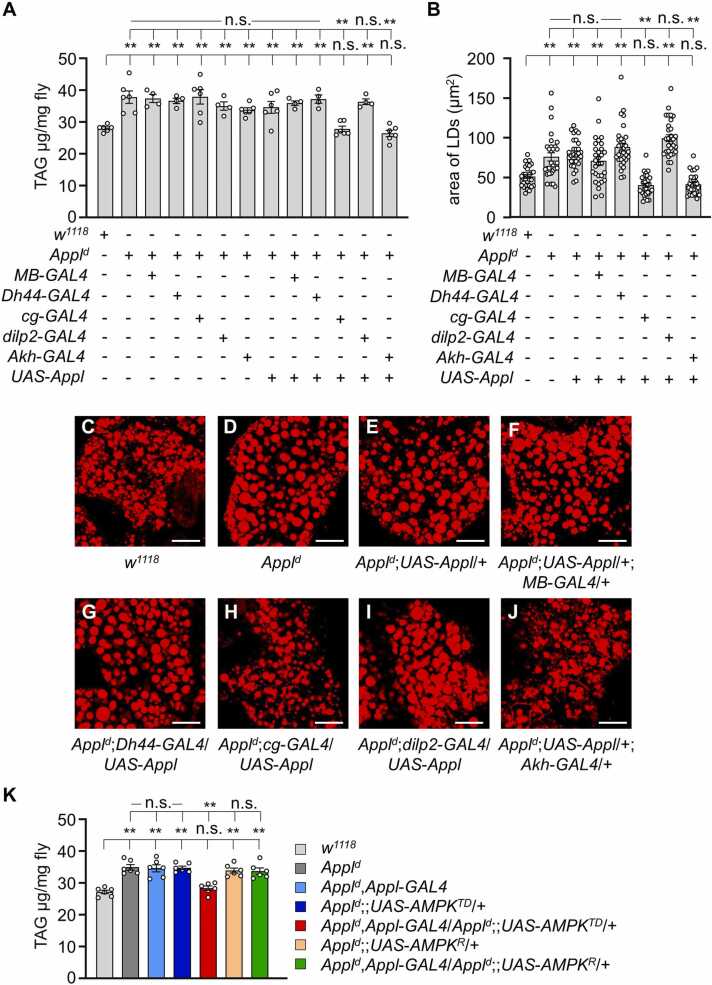
Appl expression in the fat body or adipokinetic hormone cells recapitulates the excessive TAG level in *Appl*^*d*^. (A) Measurement of TAG level in adult males of the indicated genotypes (*n* = 4-6). (B) Area of lipid droplets in the FB from (C-J) (*n* = 3). (C-J) Nile red staining of lipid droplets in the fat body of *w*^*1118*^, *Appl*^*d*^, *Appl*^*d*^;*UAS*-*Appl*, *Appl*^*d*^;*UAS*-*Appl*/+;*MB*-*GAL4*/+, *Appl*^*d*^;*Dh44*-*GAL4*/*UAS*-*Appl*, *Appl*^*d*^;*CG*-*GAL4*/*UAS*-*Appl*, *Appl*^*d*^;*diLP2*-*GAL4*/*UAS*-*Appl*, and *Appl*^*d*^;*UAS*-*Appl*/+;*AKH*-*GAL4*/+. Scale bars = 50 µm. (K) Measurement of TAG levels in adult males of *w*^*1118*^, *Appl*^*d*^, *Appl*^*d*^,*Appl*-*GAL4*, *Appl*^*d*^;;*UAS*-*AMPK*^*TD*^/+, *Appl*^*d*^,*Appl*-*GAL4*/*Appl*^*d*^;;*UAS*-*AMPK*^*TD*^/+, *Appl*^*d*^;;*UAS*-*AMPK*^*R*^/+, *Appl*^*d*^,*Appl*-*GAL4*/*Appl*^*d*^;;*UAS*-*AMPK*^*R*^/+ (*n* = 6). All values are reported as means ± SEM. Comparisons between multiple experimental groups were conducted using single-factor ANOVA with Scheffe’s post hoc test. The asterisks indicate significant differences from the controls or the indicated genotypes (**P < .01). Each dot represents the distribution of individual sample values. (+) and (−) denote the presence or absence of the indicated transgenes, respectively.

To test whether the defects in lipid deposition could be rescued, we expressed the wild-type *Appl* transgene under the control of *Appl*-*GAL4*. This procedure successfully restored TAG levels to near-normal values (Fig. 2F). We also examined LDs in the rescued line by Nile red staining (Fig. 2G-I). Quantification of the LD areas confirmed that the defect was restored to normal levels (Fig. 2J). We further investigated whether the lipid metabolic alterations in *Appl*^*d*^ flies were due to changes in lipolysis or lipogenesis by quantifying the expression of lipogenic and lipolytic genes (Fig. 2K). The levels of 2 major lipogenic enzymes, *acetyl coenzyme A carboxylase* (*acc*) and *desaturase 1* (*desat1*), were found to be normal compared with the controls (Fig. 2K). Similarly, the level of the major lipolytic enzyme *brummer* (*bmm*) lipase was comparable to that in control flies. These findings suggest that APPL-associated lipid accumulation may involve other internal mechanisms beyond simple changes in lipogenesis or lipolysis. To narrow down the tissues where APPL controls lipid accumulation, we investigated several GAL4 lines to rescue the defects in *Appl*^*d*^ flies. Specifically, we expressed the *UAS*-*Appl* transgene under the control of various GAL4 lines in the *Appl*^*d*^ mutant background, including FB-specific *cg*-*GAL4* (Lee et al., 2018a), *diuretic hormone 44*–specific *Dh44*-*GAL4* (Jang et al., 2017), *drosophila insulin-like peptide 2*–specific *dilp2*-*GAL4* (Géminard et al., 2009), and *adipokinetic hormone*–specific *Akh*-*GAL4* (Sehadova et al., 2020). Although we recently reported that *Dh44*-*GAL4* can recover *trpγ* lipid defects (Nath et al., 2024), we found that only the FB-specific *cg*-*GAL4* and *Akh*-*GAL4* lines successfully restored the elevated TAG levels in *Appl*^*d*^ flies to normal levels, whereas *Dh44*-*GAL4* and *dilp2*-*GAL4* did not (Fig. 3A). To further support our findings, we performed Nile red staining of lipids in the FBs for these specific fly lines and measured the area of LDs. Our findings revealed that only *cg*-*GAL4* and *Akh*-*GAL4* decreased the enlarged LDs to normal size, whereas *Dh44*-*GAL4* and *dilp2*-*GAL4* did not (Fig. 3B-J).

AMP-activated protein kinase (AMPK) is essential in *Drosophila* and participates in a variety of critical functions throughout the animal kingdom (Sinnett and Brenman, 2016). As the primary regulator of cellular energy balance, AMPK senses energy shortages, often indicated by a high AMP-to-ATP ratio (Herzig and Shaw, 2018). When activated, AMPK initiates adaptive responses to preserve energy homeostasis, enhancing energy-generating pathways such as glucose uptake and fatty acid oxidation, while suppressing energy-consuming activities such as protein synthesis. To investigate the role of AMPK in lipid metabolism in *Appl*^*d*^ flies, we expressed AMPK driven by *Appl*-*GAL4* in both a constitutively activated form (*UAS*-*AMPK*^*TD*^) and an inactivated form (*UAS*-*AMPK*^*R*^). Only the activated *UAS*-*AMPK*^*TD*^ restored the elevated TAG levels in *Appl*^*d*^ flies to normal levels, whereas the inactivated form did not (Fig. 3K). To further examine whether AMPK activation enhances the *Appl* expression level, we performed qRT-PCR; however, quantification data showed that AMPK did not affect the *Appl* expression levels (Fig. S2). Overall, these data suggest that APPL mutation leads to alterations in energy homeostasis, specifically affecting lipid metabolism.

### Metformin Supplementation Reduces Excessive TAG Level

**Fig. 4:**
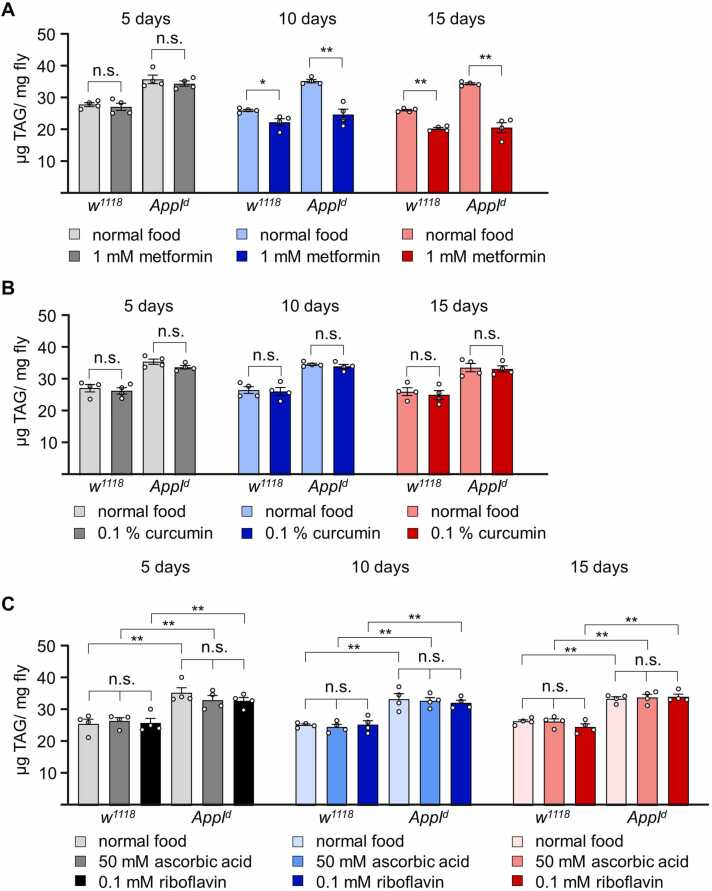
Measurement of TAG level after feeding metformin, curcumin, ascorbic acid, and riboflavin. (A) TAG levels in *w*^*1118*^ and *Appl*^*d*^ after feeding 1 mM metformin for 5, 10, and 15 days (*n* = 4). (B) TAG levels in *w*^*1118*^ and *Appl*^*d*^ after feeding 0.1% curcumin for 5, 10, and 15 days (*n* = 4). (C) Measurement of TAG level in *w*^*1118*^ and *Appl*^*d*^ after feeding 50 mM ascorbic acid and 0.1 mM riboflavin for 5, 10, and 15 days (*n* = 4). All values are reported as means ± SEM. Comparisons between 2 groups (A and B) were conducted using Student’s t-test. Comparisons between multiple experimental groups were conducted using single-factor ANOVA with Scheffe’s post hoc test. Asterisks indicate significant differences from the controls (**P < .01). Each dot represents the distribution of individual sample values.

Metformin supplementation reduces excessive TAG levels through the stimulation of AMPK (Zhou et al., 2001). In our previous study, we found that metformin helps reduce excess TAG levels in *Drosophila* (Nath et al., 2024). Here, we supplemented standard cornmeal food with 1 mM metformin (Nath et al., 2024, Sang et al., 2021) and fed it to control and *Appl*^*d*^ flies for 5, 10, and 15 days (Fig. 4A). After 5 days of feeding with 1 mM metformin, there was no significant reduction in TAG levels in control and *Appl*^*d*^ flies (Fig. 4A). However, after 10 and 15 days of metformin supplementation, there was a significant reduction in excessive TAG levels in both control and *Appl*^*d*^ flies (Fig. 4A). Specifically, 10 days of metformin supplementation reduced TAG levels from 26.34 ± 0.34 to 22.42 ± 1.12 µg/mg fly in control flies and from 35.39 ± 0.52 to 24.86 ± 1.68 µg/mg fly in *Appl*^*d*^ flies. Similarly, 15 days of metformin supplementation reduced TAG levels from 26.23 ± 0.27 to 20.43 ± 0.39 µg/mg fly in control flies, and from 34.55 ± 0.31 to 20.69 ± 1.59 µg/mg fly in *Appl*^*d*^ flies (Fig. 4A). Next, we tested the effects of 0.1% curcumin supplementation, feeding the flies for 5, 10, and 15 days. TAG levels were measured after each period, revealing that 0.1% curcumin did not reduce excessive TAG levels at any of the time points tested (Fig. 4B). Additionally, we tested the effects of 50 mM ascorbic acid and 0.1 mM riboflavin supplementation for 5, 10, and 15 days. Neither ascorbic acid nor riboflavin reduced TAG levels at any of the time points tested (Fig. 4C). These data suggest that only metformin can reduce the lipid levels in control and *Appl*^*d*^ flies, whereas curcumin, ascorbic acid, and riboflavin do not have the same effect. This difference highlights metformin’s specific role in modulating lipid metabolism in *Drosophila*, likely through AMPK activation, and suggests it may be uniquely beneficial for managing lipid dysregulation.

### The Taste-associated Memory Defects in *Appl*^*d*^ Can Be Rescued by Expressing the Wild-type *Appl* in the MB and by Curcumin Supplementation

**Fig. 5:**
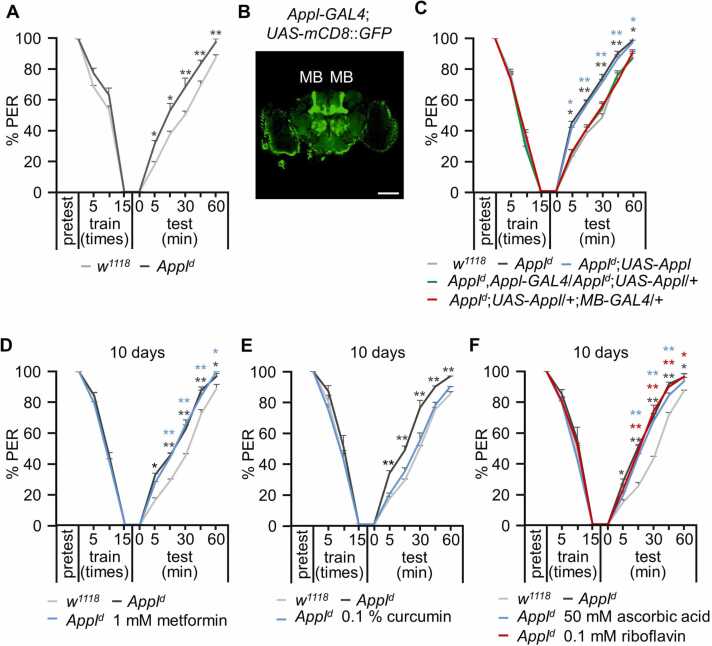
Appl mutant exhibits defects in taste-associative learning and memory, which can be recovered by expressing *Appl* in the MB or feeding curcumin. (A) Associative taste memory assay in *w*^*1118*^ and *Appl*^*d*^ (*n* = 4). (B) Expression pattern of APPL (*Appl*-*GAL4*;*UAS*-*mCD8*::*GFP*) in the brain, specifically in the mushroom body. Scale bar = 100 µm. (C) Recovery of taste-associated memory of *Appl*^*d*^ by expressing the wild-type *Appl* transgene using *Appl*-*GAL4* or *MB*-*GAL4* (*n* = 5). (D) Taste-associative learning and memory in *w*^*1118*^, *Appl*^*d*^, and *Appl*^*d*^ fed 1 mM metformin for 10 days (*n* = 4). (E) Taste-associative learning and memory in *w*^*1118*^, *Appl*^*d*^, and *Appl*^*d*^ fed 0.1% curcumin for 10 days (*n* = 4). (F) Taste-associative learning and memory in *w*^*1118*^, *Appl*^*d*^, and *Appl*^*d*^ fed 50 mM ascorbic acid or 0.1 mM riboflavin for 10 days (*n* = 4). All values are reported as means ± SEM. Comparisons between multiple experimental groups were conducted using single-factor ANOVA with Scheffe’s post hoc test. The asterisks indicate significant differences from the controls (*P < .05, **P < .01).

Various studies on *Drosophila* have demonstrated that the *Appl*^*d*^ mutant exhibits alterations in various forms of memory, such as medium-term memory and long-term memory (Bourdet et al., 2015, Goguel et al., 2011). In this study, we conducted a taste-associative learning and memory assay and found that the *Appl*^*d*^ mutant showed defects in taste-associative memory for up to 1 hour but did not exhibit learning process defects (Fig. 5A). Previous studies have demonstrated the expression of APPL in the MBs (Bourdet et al., 2015). Therefore, we conducted immunostaining and imaging for APPL expression in the brain and found that APPL is broadly expressed in various tissues, including in the MBs (Fig. 5B). To confirm the memory defect in *Appl*^*d*^, we rescued it by expressing a wild-type APPL transgene driven by *Appl*-*GAL4* (Fig. 5C). MBs are linked to various functions, such as memory, learning, and sensory integration (Sgammeglia and Sprecher, 2022). Therefore, we conducted a memory assay to rescue the *Appl*^*d*^ memory defect using *MB*-*GAL4*, which successfully restored the memory function (Fig. 5C). Although aging is not the sole cause of AD, the risk increases with age, with most cases occurring in individuals over 65. In this study, memory assays were conducted in 15- and 30-day-old flies. In control flies, associative taste memory was normal at both ages (Fig. S3A), whereas memory defects worsened with age in *Appl*^*d*^ flies (Fig. S3B).

Next, learning and memory assays were conducted after feeding flies metformin, curcumin, ascorbic acid, and riboflavin for 5, 10, and 15 days. Metformin supplementation did not recover the altered taste-associative memory in *Appl*^*d*^ flies (Figs. S3C, 5D, and S3D). Feeding with 0.1% curcumin for 5 days did not improve the memory defect in *Appl*^*d*^ flies (Fig. S3E). However, feeding with 0.1% curcumin for 10 and 15 days did improve memory in *Appl*^*d*^ flies (Figs. 5E and S3F). The fly food was also supplemented with vitamins, specifically vitamin C (50 mM ascorbic acid) and vitamin B_2_ (0.1 mM riboflavin), for 5, 10, and 15 days, after which learning and memory assays were conducted. Neither ascorbic acid nor riboflavin recovered the taste-associative memory defect in *Appl*^*d*^ flies at any tested time period (Figs. S3G, 5F, and S3H). These data suggest that curcumin can enhance memory impairment in *Appl*^*d*^ flies, whereas metformin, ascorbic acid, and riboflavin do not have this effect.

### *Appl*^*d*^ Mutants Exhibit Reduced Climbing Ability, Which Can Be Improved by Expressing *Appl* in the MB and Muscle, as Well as by Vitamin Supplementation

**Fig. 6:**
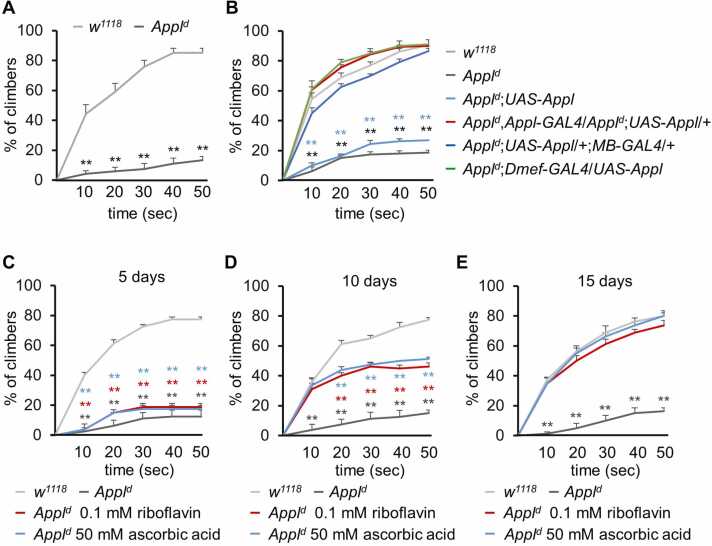
Appl mutant exhibits defects in climbing ability, which can be recovered by expressing *Appl* in MBs or muscle cells and by feeding vitamin C or B_2_. (A) Measurement of climbing ability in *w*^*1118*^ and *Appl*^*d*^ (*n* = 6). (B) Recovery of climbing defect of *Appl*^*d*^ by expressing the wild-type *Appl* transgene using *Appl*-*GAL4*, *MB*-*GAL4*, and *Dmef*-*GAL4* (*n* = 5-8). (C-E) Measurement of climbing ability in *w*^*1118*^, *Appl*^*d*^, and *Appl*^*d*^ fed ascorbic acid and riboflavin for 5, 10, and 15 days (*n* = 4). All values are reported as means ± SEM. Comparisons between multiple experimental groups were conducted using single-factor ANOVA with Scheffe’s post hoc test. The asterisks indicate significant differences from the controls (**P < .01).

Climbing is a crucial behavior in insects, allowing them to find food sources, escape predators, locate mates, and navigate their surroundings. In this study, we measured the climbing ability of control and *Appl*^*d*^ flies for 50 seconds and found that the climbing ability of *Appl*^*d*^ mutants was 4 times lower than the control (Fig. 6A). To determine if the climbing defect in *Appl*^*d*^ could be rescued, we expressed *UAS*-*Appl* under the control of *Appl*-*GAL4*. This successfully restored the climbing ability in *Appl*^*d*^ flies (Fig. 6B). Although MBs are not directly linked to motor function, they can modulate dopaminergic neurons and Kenyon cells, affecting locomotor responses and startle-induced movements. Here, climbing ability was tested by expressing *UAS*-*Appl* under *MB*-*GAL4* control, which also rescued the climbing defect in *Appl*^*d*^ flies (Fig. 6B). Coordinated muscle contractions generate the rhythmic movements required for climbing. To investigate whether muscle-specific GAL4 could recover the *Appl*^*d*^-associated climbing defect, climbing ability was measured using the muscle-specific *Dmef*-*GAL4* (Lee et al., 2018a). This approach successfully restored the climbing ability in *Appl*^*d*^ flies (Fig. 6B), indicating that Appl plays a crucial role in MB and muscle tissues to coordinate movement.

Next, we explored potential candidates to enhance climbing ability in *Appl*^*d*^ flies by supplementing standard cornmeal food with 1 mM metformin for 5, 10, and 15 days. Metformin feeding did not recover the climbing impairments in *Appl*^*d*^ flies (Fig. S4A-C). Similarly, feeding flies with 0.1% curcumin for 5, 10, and 15 days did not improve the impaired climbing ability of *Appl*^*d*^ flies (Fig. S4D-F). However, while feeding with ascorbic acid and riboflavin did not improve climbing defects in 5 days (Fig. 6C), both vitamins enhanced climbing ability after 10 and 15 days (Fig. 6D and E). Collectively, our data suggest that ascorbic acid and riboflavin can enhance the climbing ability of *Appl*^*d*^ flies.

## DISCUSSION

**Fig. 7:**
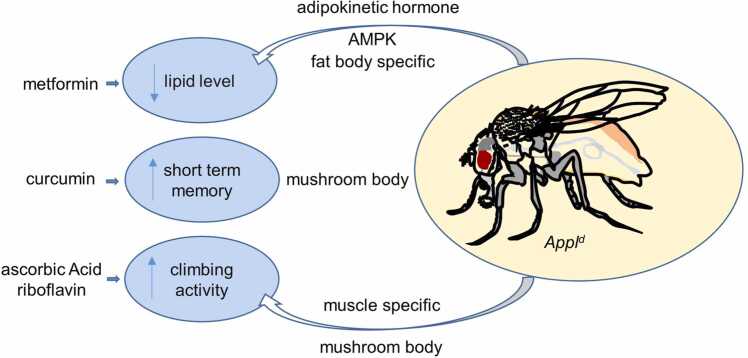
Schematic model illustrating the involvement of *Appl* in the regulation of lipid metabolism, cognitive functions, and climbing activity and possible genetic and therapeutic reagents that can ameliorate *Appl*^*d*^-associated defects.

Our findings provide insights into the crucial roles of APPL in regulating lipid metabolism, memory formation, and motor functions in *Drosophila*. Particularly, our results indicated that APPL deficiency leads to significant metabolic and physiological impairments, which can be mitigated by targeted genetic and dietary interventions (Fig. 7).

APPL mutation resulted in a reduced lifespan, increased TAG levels, and enlarged LDs in the FB. This underscores APPL's critical role in lipid homeostasis. Notably, *Appl*^*d*^ mutants displayed resistance to starvation, suggesting that *Appl*^*d*^ retains normal lipid utilization function. However, *Appl*^*d*^ mutants had significantly shorter lifespans compared with controls. We also observed age-dependent degeneration in *Appl*^*d*^ flies, such as reduced climbing ability and short-term memory defects. Loss of APPL was associated with dysregulated translation, impaired mitochondrial function, and disrupted lipid metabolism, based on multiple subnetwork analyses (Nithianandam et al., 2023). These findings suggest that autophagy regulation and vacuole formation in the brain may influence the lifespan of *Appl*^*d*^ mutants. Although there is no direct evidence that APPL affects mitochondrial function, metabolic characterization of intact cells has shown that intracellular amyloid beta, rather than APP, reduces mitochondrial respiration (Schaefer et al., 2016). Further studies are needed to verify any functional impact on mitochondria in relation to lipid metabolism.

Genetic rescue experiments demonstrated that expressing wild-type APPL under the control of *Appl*-*GAL4* or FB-specific *cg*-*GAL4* and *Akh*-*GAL4* successfully normalized TAG levels and LD size. Conversely, *Dh44*-*GAL4* and *dilp2*-*GAL4* drivers did not restore lipid homeostasis, highlighting the specific tissue and hormonal pathways through which APPL operates. AMPK, a pivotal energy sensor (Hardie and Ashford, 2014), was shown to be essential in this context. Expressing a constitutively active form of AMPK (*UAS*-*AMPK*^*TD*^) under *Appl*-*GAL4* reduced excessive TAG levels, whereas an inactive form of AMPK did not. This suggests that AMPK activation is a key downstream effect of APPL in maintaining lipid balance. Our findings align with the known metabolic functions of AMPK, which enhances energy-generating pathways and suppresses energy-consuming processes under low-energy conditions.

Metformin, a widely prescribed diabetes medication, significantly reduced excessive TAG levels in *Appl*^*d*^ flies after 10 and 15 days of supplementation. Metformin’s efficacy likely stems from its ability to activate AMPK-mediated pathways, promoting lipolysis and improving energy homeostasis. In contrast, neither curcumin, ascorbic acid, nor riboflavin supplementation significantly affected TAG levels, indicating their limited role in lipid regulation under the conditions tested.

Previous studies have reported long-term memory impairment in *Appl*^*d*^ flies. Our study further demonstrated that *Appl*^*d*^ mutants exhibit short-term associative taste memory defects, which can be rescued by expressing wild-type APPL in the MBs. Curcumin supplementation also improved memory defects, specifically after 10 and 15 days of feeding. Curcumin is known for its neuroprotective effects, including reducing oxidative stress, enhancing brain-derived neurotrophic factors, and preventing amyloid-beta aggregation (Mishra and Palanivelu, 2008, Small et al., 2018), which may explain its positive impact on memory.

APPL deficiency significantly impairs climbing ability, a crucial trait for insect survival and behavior. Genetic rescue using *Appl*-*GAL4*, *MB*-*GAL4*, and muscle-specific *Dmef*-*GAL4* restored climbing ability in *Appl*^*d*^ flies, suggesting the involvement of APPL in both central and peripheral motor functions. Although metformin and curcumin did not improve climbing defects, supplementation with ascorbic acid and riboflavin for 10 and 15 days did enhance motor performance. Vitamin C is a potent antioxidant that scavenges free radicals and shields brain cells from oxidative stress (Traber and Stevens, 2011). Oxidative stress is one of the factors in the pathogenesis of AD. Patients with AD exhibit signs of nuclear and mitochondrial oxidation in the parietal cortex, as well as lipid peroxidation in the temporal cortex. Similarly, riboflavin protects against neurotoxicity by ameliorating oxidative stress, mitochondrial dysfunction, neurogenic inflammation, and glutamate excitotoxicity (Marashly and Bohlega, 2017). Through its cofactors FMN and FAD, riboflavin controls the structure and function of flavoenzymes, protecting cells from oxidative stress and apoptosis (Udhayabanu et al., 2017). These vitamins are known antioxidants and neuroprotectants (Chambial et al., 2013, Plantone et al., 2021), which likely mitigate oxidative stress and support neuronal health.

Our findings highlight the multifaceted role of APPL in *Drosophila*, encompassing metabolic regulation, cognitive functions, and motor abilities. The different effects of genetic and dietary interventions provide insights into potential therapeutic targets for metabolic and neurodegenerative disorders. Metformin’s impact on lipid metabolism suggests its potential utility in treating obesity, while curcumin's effects on memory imply its relevance for AD. The positive outcomes of vitamin supplementation on motor functions serve as a basis for the development of dietary strategies to support neuronal health. Future studies should thus aim to unravel the precise molecular mechanisms underlying APPL’s functions and the pathways through which metformin and curcumin exert their effects. Understanding these mechanisms may pave the way for novel treatments for metabolic and neurodegenerative diseases.

## Author Contributions

D.K.N. performed the experiments, analyzed the data, generated the figures, and wrote the original draft. Y.L. supervised, reviewed, and edited the draft, wrote the original paper, and obtained the funding.

## Declaration of Competing Interests

The authors have no financial interest.

## Footnotes

Appendix A Supplemental material associated with this article can be found online at: doi:10.1016/j.mocell.2024.100163.
