# Supplementary material for: Exploring the multifaceted functions of APPL in metabolism and memory using Drosophila melanogaster
Source: Mol Cells. 2024 Nov 26;48(1):100163. doi: 10.1016/j.mocell.2024.100163 (PMC11697555; doi:10.1016/j.mocell.2024.100163)
Supplement: Supplementary file 1 — Supplementary material [file mmc1.pdf]

Supplememntary figures

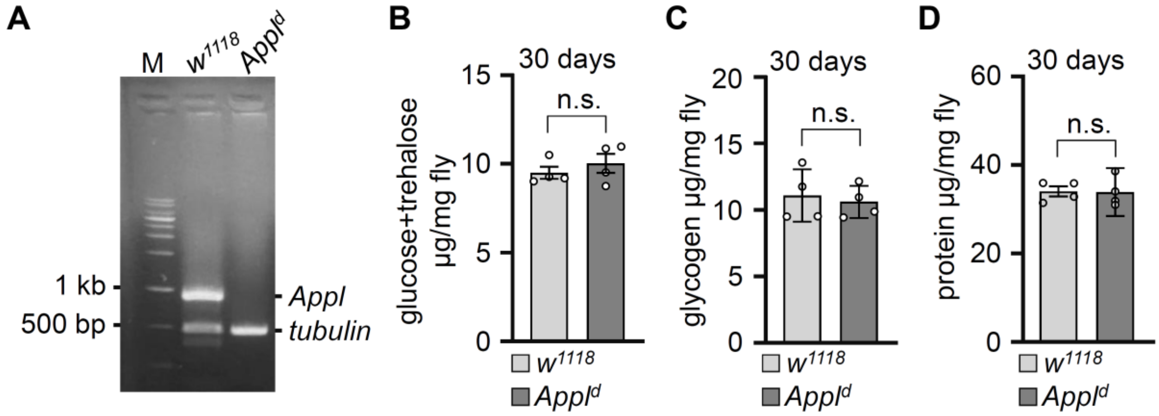

Fig. S1 Confirmation of *Appl*^{d} null mutation. (A) Gel picture of RT-PCR results showing the expression of *Appl* in wild type (*w*^{1118}) flies but not in *Appl*^{d} mutant flies, M represent the 1 kb (+) marker. The predicted size of *Appl* is 886 bp and *tubulin* was used as an internal control for PCR reaction of cDNA. (B) Measurement of whole body glucose and trehalose level in 30 day old *w*^{1118} and *Appl*^{d} flies (n=4). (B) Measurement of whole body glycogen level in 30 day old *w*^{1118} and *Appl*^{d} flies (n=4). (C) Measurement of whole body protein level in 30 day old *w*^{1118} and *Appl*^{d} flies (n=4).

All values are presented as means ± SEM. Comparisons between multiple experimental groups were performed using single-factor ANOVA followed by Scheffe’s *post hoc* test.

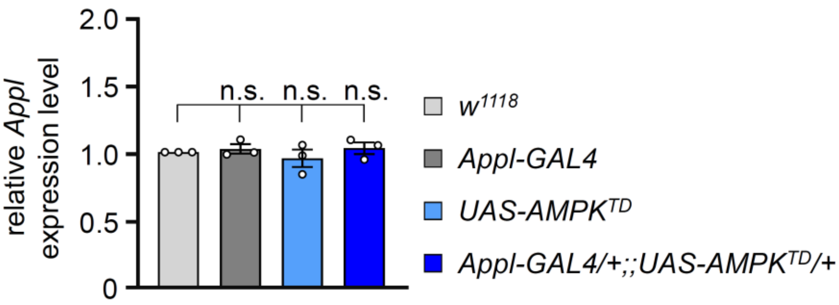

Fig. S2. qRT-PCR analyses for the measurement of *Appl* expression level in *w*^{1118}, *Appl-GAL4*, *UAS-AMPK*^{TD}, and *Appl-GAL4*/+;;UAS-AMPK^{TD}/+. (n=3).

All values are presented as means ± SEM. Comparisons between multiple experimental groups were performed using single-factor ANOVA followed by Scheffe's *post hoc* test.

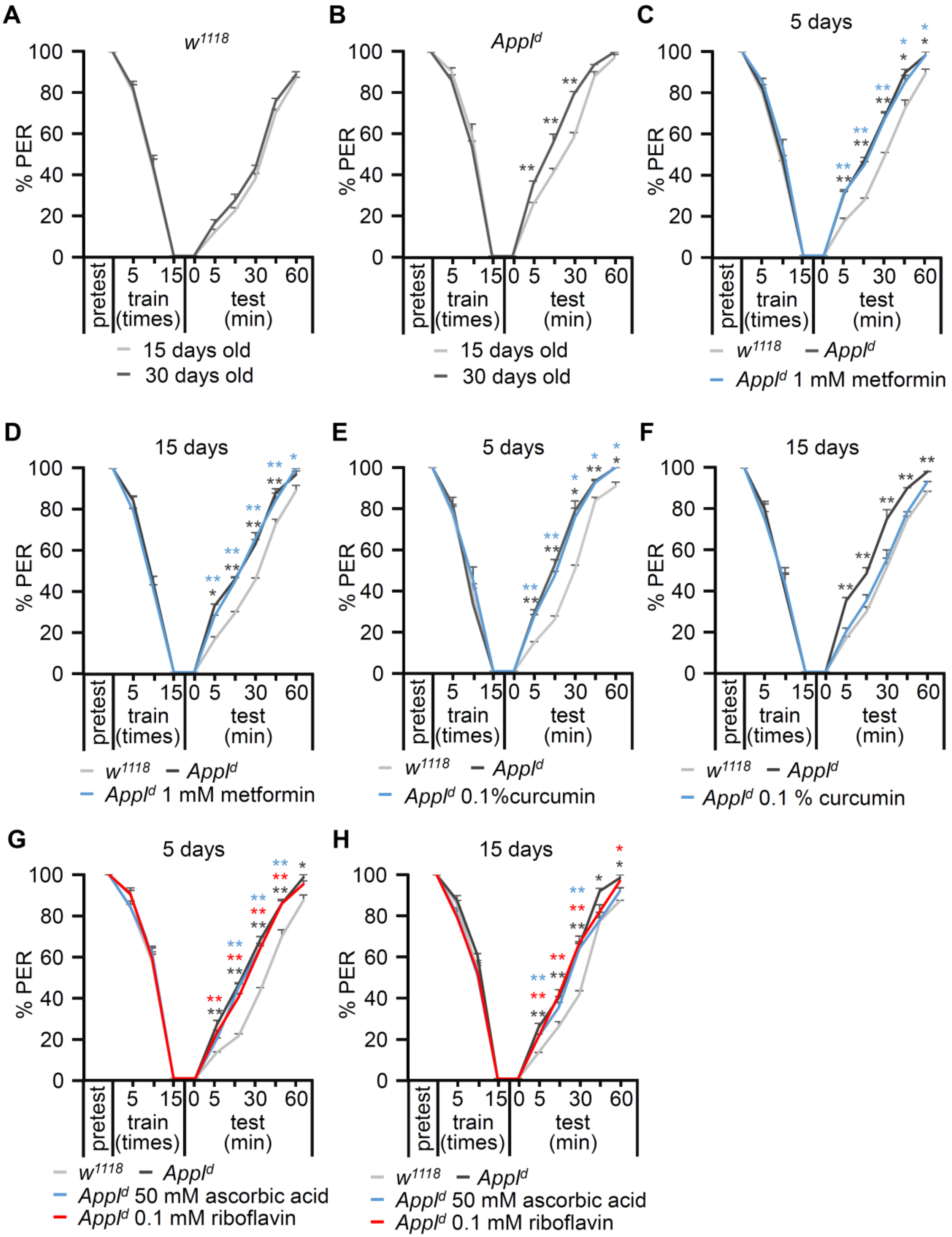
A
w1118
B
Appld
C
5 days
D
15 days
E
5 days
F
15 days
G
5 days
H
15 days
% PER
100
80
60
40
20
0
pretest
5
15
train
(times)
0
5
30
60
test
(min)
15 days old
30 days old
w1118
Appld
Appld 1 mM metformin
Appld 0.1%curcumin
Appld 0.1 % curcumin
Appld 50 mM ascorbic acid
Appld 0.1 mM riboflavin

Fig. S3. Learning and memory assay after feeding metformin, curcumin, ascorbic acid, and riboflavin. (A) Associative taste memory assay for w^{1118} with 15 days and 30 days old flies (n=4–5). (B) Associative taste memory assessment for $Appl^{d}$ with 15 days and 30 days old flies (n=4–5). (C) Taste associative learning and memory for w^{1118}, $Appl^{d}$, and $Appl^{d}$ fed 1 mM metformin for 5 days (n=4). (D) Taste associative learning and memory for w^{1118}, $Appl^{d}$, and $Appl^{d}$ fed 1 mM metformin for 15 days (n=4). (E) Taste associative learning and memory for w^{1118}, $Appl^{d}$, and $Appl^{d}$ fed 0.1 % curcumin for 5 days (n=4). (F) Taste associative learning and memory for w^{1118}, $Appl^{d}$, and $Appl^{d}$ fed 0.1 % curcumin for 15 days (n=4). (G) Taste associative learning and memory for w^{1118}, $Appl^{d}$, and $Appl^{d}$ fed 50 mM ascorbic acid and 0.1 mM riboflavin for 5 days (n=4). (H) Taste associative learning and memory for w^{1118}, $Appl^{d}$, and $Appl^{d}$ fed 50 mM ascorbic acid and 0.1 mM riboflavin for 15 days (n=4).

All values are reported as means ± SEM. Comparisons between multiple experimental groups were conducted via single-factor ANOVA coupled with Scheffe's *post hoc* test. The asterisks indicate significant differences from the controls (**P*<0.05, ***P*<0.01).

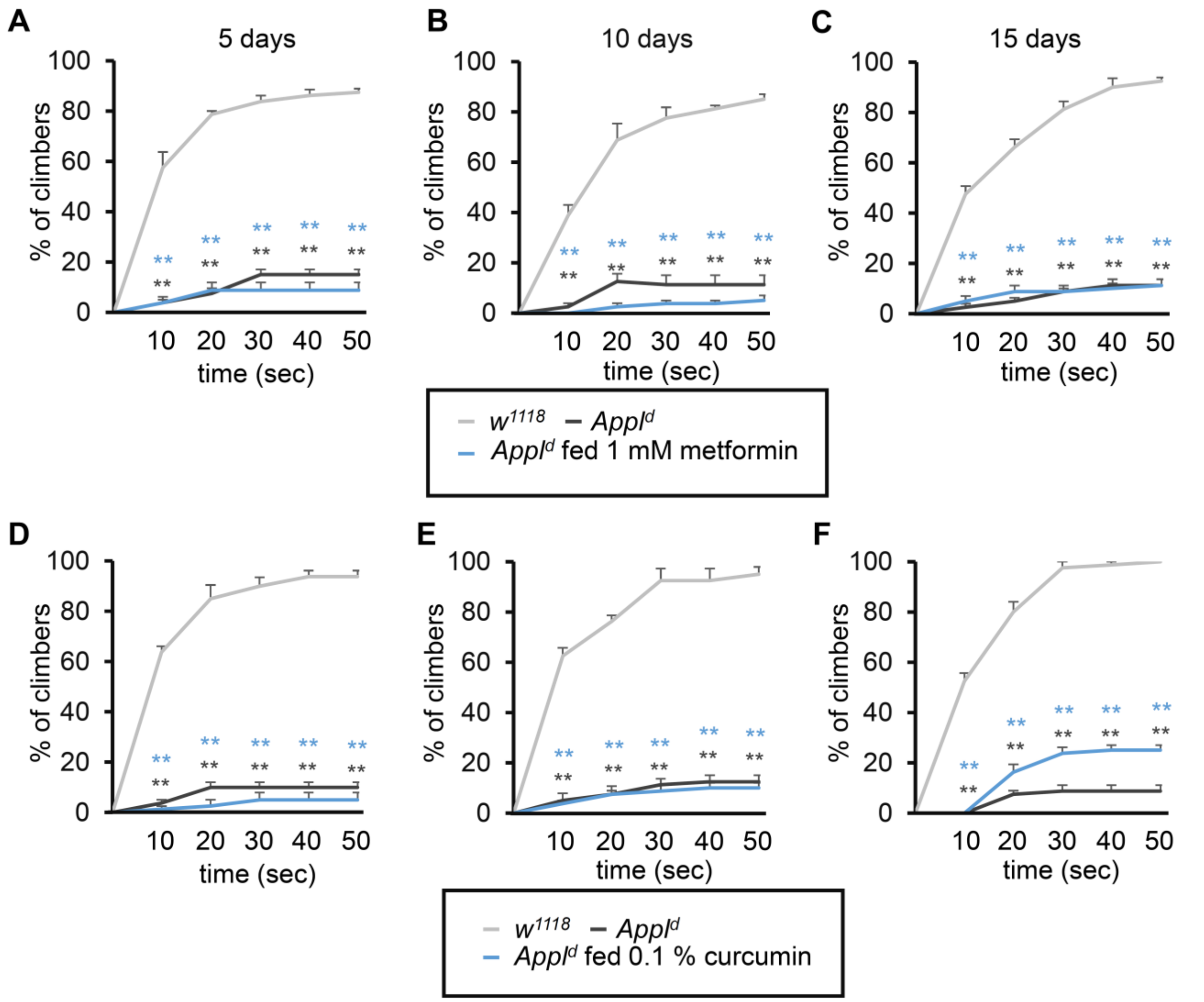

Fig. S4. Climbing ability measurement after feeding metformin and curcumin. (A) Measurement of climbing ability in w^{1118}, $Appl^d$, and $Appl^d$ fed 1 mM metformin for 5 days (n=4). (B) Measurement of climbing ability in w^{1118}, $Appl^d$, and $Appl^d$ fed 1 mM metformin for 10 days (n=4). (C) Measurement of climbing ability in w^{1118}, $Appl^d$, and $Appl^d$ fed 1 mM metformin for 15 days (n=4). (D) Measurement of climbing ability in w^{1118}, $Appl^d$, and $Appl^d$ fed 0.1 % curcumin for 5 days (n=4). (E) Measurement of climbing ability in w^{1118}, $Appl^d$, and $Appl^d$ fed 0.1% curcumin for 10 days (n=4). (F) Measurement of climbing ability in w^{1118}, $Appl^d$, and $Appl^d$ fed 0.1 % curcumin for 15 days (n=4).

All values are reported as means ± SEM. Comparisons between multiple experimental groups were conducted via single-factor ANOVA coupled with Scheffe’s *post hoc* test. The asterisks indicate significant differences from the controls (**$P<0.01$).
